# Elevated Secretion of Aldosterone Increases TG/HDL-C Ratio and Potentiates The Ox-LDL-Induced Dysfunction of HUVEC 

**DOI:** 10.22074/cellj.2021.7033

**Published:** 2021-03-01

**Authors:** Qian Zhang, Yiwen Pan, Xiaochun Ma, Hao Yang, Jun Chang, Ling Hong, Huiwen Yan, Shubing Zhang

**Affiliations:** 1. Department of Cardiovascular Surgery, Shandong Provincial Hospital Affiliated to Shandong First Medical University, Jinan, Shandong, China; 2. Department of Cell Biology, School of Life Sciences, Central South University, Changsha, Hunan, China; 3. Hunan Key Laboratory of Animal models for Human Diseases, Central South University, Changsha, Hunan, China; 4. Breast Cancer Research Center, School of Life Sciences, Central South University, Changsha, Hunan, China

**Keywords:** Aldosterone, Atherosclerosis, Human Umbilical Vein Endothelial Cells, Triglyceride/High-Density Lipoprotein Cholesterol, Oxidized Low-Density Lipoprotein

## Abstract

**Objective:**

Atherosclerosis (AS) is one of the most common causes of human death and disability. This study is designed to investigate the roles of aldosterone (Aldo) and oxidized low-density lipoprotein (Ox-LDL) in this disease by clinical data and cell model.

**Materials and Methods:**

In this experimental study, clinical data were collected to investigate the Aldo role for the patients with primary aldosteronism or adrenal tumors. Cell viability assay, fluorescence-activated cell sorting (FACS) assay, apoptosis assay, cell aging analysis, and matrigel tube formation assay were performed to detect effects on human umbilical vein endothelial cells (HUVECs) treated with Aldo and/or Ox-LDL. Quantitative polymerase chain reaction (qPCR) and Western blot analysis were performed to figure out critical genes in the process of endothelial cells dysfunction induced by Aldo and/or Ox-LDL.

**Results:**

We found that the Aldo level had a positive correlation with the TG/HDL-C ratio. Endothelial cell growth, angiogenesis, senescence, and apoptosis were significantly affected, and eNOS/Sirt1, the value of Bcl-2/Bax and Angiopoietin1/2 were significantly affected when cells were co-treated by Aldo and Ox-LDL.

**Conclusion:**

Elevated Aldo with high Ox-LDL together may accelerate the dysfunction of HUVEC, and the Ox-LDL, especially for those patients with high Aldo should be well controlled. The assessment of the role of Aldo may provide a theoretical basis for the effective prevention and investigation of a new treatment of AS.

## Introduction

Atherosclerosis (AS) is a chronic immune-inflammatory disease characterized by abnormal lipid metabolism in the arterial wall, which causes a variety of cardiovascular and cerebrovascular diseases and is one of the most common causes of human death and disability (1). Aldosterone (Aldo), the main mineralocorticoid hormone, belongs to the Renin-angiotensin-aldosterone-system and may play an important role in vascular injury and remodeling (2, 3). Excessive Aldo will cause hypertension, hypokalemia, vascular inflammation, injury, loss of vascular function, and the initiation and progression of AS (4, 5). In patients with known AS, higher Aldo levels predict a substantially increased risk of cardiovascular death, but the mechanisms are poorly understood (6).

Animal studies demonstrated that Aldo functions in atherosclerotic plaque formation, and Aldo infusion increased overall aortic plaque area with enhanced oxidative stress (7). Clinical trials showed that inhibition of Aldo production or inhibition of the mineralocorticoid receptor, which mediates Aldo’s effects can reduce cardiovascular ischemic events and mortality (8). Some studies suggest that Aldo can promote vascular inflammation, injury, and dysfunction. For example, nitric oxide (NO) functions as the relaxation factor of vascular smooth muscle and may have an anti-AS effect, while Aldo can reduce NO synthesis in the vascular wall (9, 10). Endothelial progenitor cells (EPC) are mainly involved in the renewal, vascular repair, and angiogenesis of VECs, while Aldo can lead to the dysfunction and damage of EPC (11). In addition, Aldo regulates vascular fibrosis and promotes insulin resistance by up-regulating insulinlike growth factor 1 receptor (IGF1R) in vascular smooth muscle cells (12). The above evidence suggests that Aldo probably function importantly in the occurrence of AS and mechanism involved need to be further clarified.

Vascular endothelial cells (VECs) dysfunction is found in the lesion-prone areas of arterial vasculature, resulting in the earliest detectable changes in the process of an atherosclerotic lesion (13). This leads into a complex pathogenic sequence, initially involving the selective recruitment of monocytes into the intima, where they differentiate into macrophages and become foam cells; the released growth factors and chemokines then induce neighboring smooth muscle cells to proliferate and synthesize extracellular matrix components and then generate fibromuscular plaque (14, 15). During the development of AS, VECs are exposed to various damaging stimuli [such as oxidized low-density lipoprotein (OxLDL)], which trigger vascular endothelial injury (16). Angiogenesis has two kinds of functions on AS, which is induced in a hypoxic environment can be a reason to increase plaque of development and stability (17). However, it also has another function to repair damaged cells. Therefore, understanding the mechanism involved in VECs dysfunction induced by different factors will be helpful for better prevention and therapy of AS.

In this study, we analyzed the clinical data of these patients admitted to our department to explore the Aldo effects on the occurrence and progression of AS. At the same time, HUVECs were used to further explore the effect and mechanism of Ox-LDL and Aldo at the cellular level.

## Materials and Methods

### Patients’ data

In this experimental study, human data were obtained from the basic diagnosis of patients from Shandong Provincial Hospital affiliated to Shandong University from January 2018 to January 2019. Consent has been obtained from each patient after a full explanation of the purpose and nature of all procedures used. The research purposes under protocols were approved by the Ethics Committee of our hospital (NO.2017536). It is considered to be excessive if the serum Aldo level is more than 300 pg/mL depending on clinical practice endorsed by our hospital. The patients were divided into 3 subgroups (Aldo<300, 300<Aldo<600, Aldo>600) or 2 subgroups (Aldo<300, Aldo>300) in different data analysis.

### Chemicals

Aldo (ApexBio Technology, USA) was dissolved in dimethylsulfoxide (DMSO, Solarbio, Beijing, China) at 2 mM and stored at -20˚C. Ox-LDL was obtained from Yiyuan Biotechnologies (Guangzhou, China). Cell Counting Kit-8 (CCK8) was obtained from YEASEN (Shanghai, China). Hoechst 33342 Staining Kit was obtained from Bioworld Technology (Nanjing, China). Senescence β-Galactosidase Staining Kit was obtained from Beyotime Biotechnology (Beijing, China). 

### Cell culture

HUVECs were obtained from the Department of Endocrinology, Xiangya Third Hospital, Central South University. Cells were grown at 37˚C with RPMI 1640 (Hyclone, USA) medium supplemented with 100 μg/mL streptomycin, 100 U/mL penicillin (Solarbio, Beijing, China) and 10% fetal bovine serum (PAN-Biotech, Germany) in a humidified incubator of 5% CO_2_. 

### Cell counting assay kit-8 assay

Cell counting assay kit-8 (CCK8) was used to measure cell viability according to the manufacturer’s instructions. The cells were seeded at a density of 3000 cells/well in 96- well plates and then treated for 24, 48, 72, and 96 h with OxLDL (120 μg/mL) or/and Aldo (20 μM) diluted to various concentrations in complete medium, control was incubated with DMSO. After treatment, CCK-8 reagent was mixed to the medium (1: 10) at 37˚C for 2-4 hours and then measured absorbance at 490 nm using a microplate reader.

### Fluorescence-activated cell sorting assay

After treated with Ox-LDL or/and Aldo for 48 hours, the cells were collected by Trypsin digestion to prepare a single cell suspension and then centrifuged for 5 minutes at 1000 r/minutes. After washed with phosphate buffer saline (PBS, Beijing Dingguo, China) twice, the cells were fixed at 4˚C with 70% ice-ethanol overnight. Then, samples were stained with propidium iodide (PI) and measured using a flow cytometer.

### Apoptosis assay by Hoechst33342

Cell apoptosis was measured using a Hoechst 33342 Staining Kit. The cells grew in 24-well plates treated with Ox-LDL (120 μg/mL) or/and Aldo (20 µM), as previously described. The control group was incubated with DMSO. After 48 hours treatment, the cells were incubated in dilution buffer with Chromogen (the final concentration was 5 μg/mL) about 5 minutes in a dark incubator at 30˚C, and then washed with PBS for 3 minutes for 3 times. The sample was then observed with the fluorescence microscope. The percentage of Hoechst staining was calculated by counting the positively stained cells within a sample of 100 cells.

### Cell aging analysis

Cell aging was measured using a Senescence β-Galactosidase Staining Kit. HUVECs were treated with 20 μM Aldo alone or combined with 120 μg/mL of Ox-LDL for 48 hours. Cells were treated with the same amount of DMSO as control. The cells were rinsed using PBS and then fixed for 15 minutes with a fixation solution. After that, the cells were washed triplicate for 3 minutes and incubated with a staining solution overnight at 37˚C. Then，the cells were observed and counted with the ordinary light microscope. The percentage of SA-β-gal was calculated by counting the positively stained cells within a sample of 100 cells.

### Matrigel tube formation assay* in vitro*

After thawed on the ice overnight, Matrigel (Corning, USA) was added into a 96-well plate (50 µL/well) and incubated for 30 minutes at 37˚C to solidify. After trypsinized, the HUVECs were seeded into Matrigel-coated wells (2×10^4^ cells per well). The cells were incubated with Ox-LDL (120 µg/mL) or/and Aldo (20 µM) for 6-12 hours in RPMI 1640 medium. Cells were treated with the same amount of DMSO as control. After 6-12 hours at 37˚C, the tube formation in Matrigel was observed under a light microscope.

### Quantitative polymerase chain reaction experiment

Total RNA extraction, reverse transcription, and quantitative polymerase chain reaction (qPCR) were performed using TRIzol Up Plus RNA Kit (Transgene Biotech, China), FastQuant RT Kit (Tiangen, China) and SuperReal PreMix Plus (Tiangen, China), respectively. 

The primer sequences as followed:

eNOS was: 

F: 5′-GTTTGTCTGCGGCGATGTT-3′ 

R: 5′-GCGTGAGCCCGAAAATGTC-3′.

Sirt1 was: 

F: 5′-TGACTGGACTCCAAGGCCACGG-3′ 

R: 5′-TCAGGTGGAGGTATTGTTTCCGGCA-3′. 

Angiopoietin-1 was:

F: 5′-AGCGCCGAAGTCCAGAAAAC-3′

R: 5′TACTCTCACGACAGTTGCCAT-3′. 

Angiopoietin-2 was:

F: 5′-CTCGAATACGATGACTCGGTG-3′ 

R: 5′TCATTAGCCACTGAGTGTTGTTT-3′.

The relative abundance of mRNA was determined by the equation 2^-ΔCT^ (ΔCT=threshold cycle (CT_Tested Gene_-CT_GAPDH_). For each sample, data were derived from three repeats.

### Western blot analysis

The cells were treated with Aldo (20 μM), Ox-LDL (120 μg/mL), or Aldo plus Ox-LDL for 48 hours. The control group was incubated with DMSO. After lysed in RIPA buffer (Beyotime Biotechnology, China), the cells were centrifuged at 130000 × g for 10 minutes at 4˚C. Total proteins (25 µg) were resuspended in loading buffer and separated by 10% sodium dodecyl sulfate polyacrylamide gel electrophoresis (SDS-PAGE, Biosharp, China), followed by transferring onto a polyvinylidene fluoride membrane (18). The membranes were blocked with 5% nonfat dry milk and incubated with the primary antibody overnight. After incubated with secondary antibody and washed, blots were developed with the Efficient Chemiluminescence Kit (GENVIEW) and SageCapture imaging System (SAGECREATION). Primary antibodies are eNOS (1:1000; Cell Signaling Technology, No.9572), Bcl-2 (1:1000, Proteintech, No. 12789-1-AP), Bax (1:1000, Proteintech, No. 50599-2-Ig) and β-actin (1:2000, ABclonal, No. AC004). The second antibodies are Goat Anti-Mouse IgG (1:4000, ABclonal, No. AS003) and Goat Anti-Rabbit IgG (1:4000; Abbkine, No.A21020).

### Statistics

All data were presented as mean ± standard error. Statistical analysis was performed by the two-tailed Student t test, and it was considered statistically significant when the P<0.05. (GraphPad Prism).

## Results

A total of 327 patients (male 172, female 155) who had primary aldosteronism and adrenal tumors were examined retrospectively. The average age is 45.7 years old range from 16 to 77 years old. The demographic data of the cases were shown in Table 1.

**Table 1:** Demographic characteristics of cases


Characteristics	Male (n=172)	Female (n=155)

Age (Y)	44.29 ± 12.69	47.35 ± 11.60
Weight (kg)	80.06 ± 12.18	66.45 ± 10.74
Cholesterol (mmol/L)	4.88 ± 1.18	5.16 ± 1.15
HDL(mmol/L)	1.18 ± 0.28	1.40 ± 0.31
LDL (mmol/L)	4.00 ± 14.98	3.00 ± 0.94
TG (mmol/L)	1.88 ± 1.33	1.52 ± 0.88
ALD (pg/ml)	292.34 ± 255.05	289.08 ± 184.84


Data are presented as mean ± SD. HDL; High-density lipoprotein cholesterol, LDL; Low-density lipoprotein.

### Aldo level had a positive correlation with TG/HDL-C ratio

**Fig.1:**
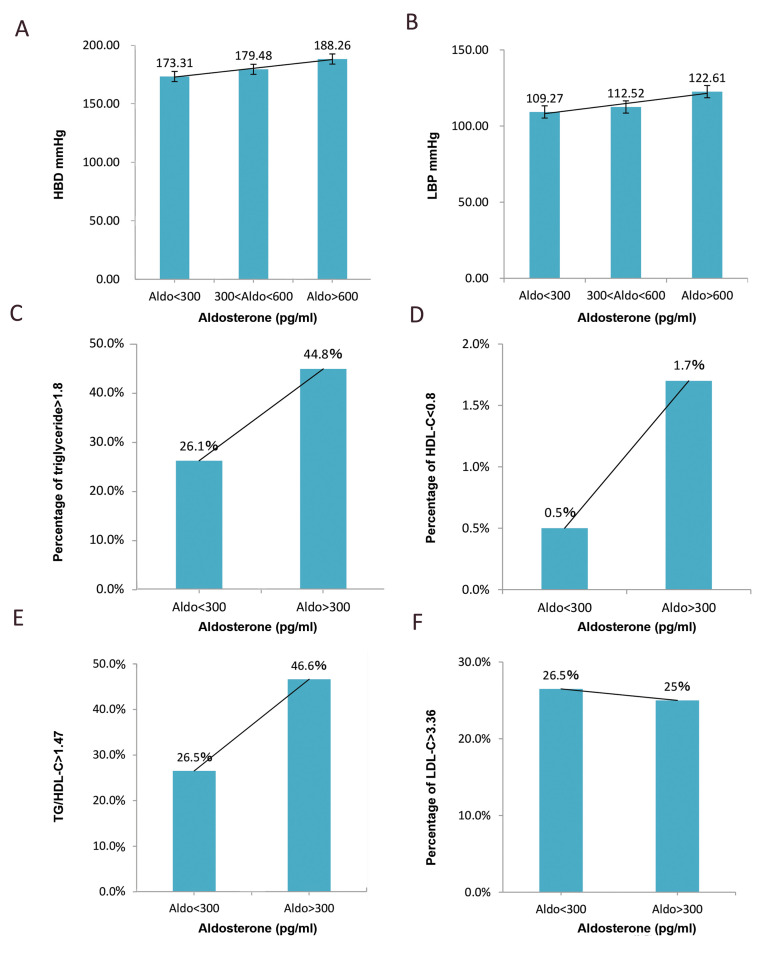
The correlation of serum Aldo level with blood pressure, TG, HDL-C, and LDL-C. The patients were divided into 3 subgroups (Aldo<300, 300<Aldo<600, Aldo>600) or 2 subgroups (Aldo<300, Aldo>300). **A. **The average vale of high blood pressure in Aldo<300, 300<Aldo<600, Aldo>600 groups. **B.** The average vale of low blood pressure in Aldo<300, 300<Aldo<600, Aldo>600 groups. **C. **The percentage of serum TG level above 1.8 mmol/L in Aldo<300 and Aldo>300 groups. **D.** The percentage of serum HDL-C level below 0.8 mmol/L in Aldo<300 and Aldo>300 groups.** E.** The percentage of TG/HDL-C ratio above the average in Aldo<300 and Aldo>300 groups.** F.** The percentage of serum LDL-C level above 3.36 mmol/L in Aldo<300 and Aldo>300 groups. TG; Triglyceride, HDL-C; High-density lipoprotein, cholesterol, LDL-C; Low-density lipoprotein, cholesterol, HBD; High blood pressure, LBP; Low blood pressure, and Aldo; Aldosterone.

Our results confirm that excessive secretion of Aldo by the adrenal cortex is characterized by hypertension (Fig.1A, B). Interestingly, the serum Aldo level is relevant to the level of TG and HDL cholesterol (HDL-C). In excessive Aldo group (Aldo>300), 44.8% of patients were beyond the high limit value of TG, while only 26.1% in the normal group (Aldo<300). In the meantime, 1.7% of patients were beyond the low limit value of HDL-C in excessive Aldo group, while only 0.5% in the normal group (Fig .1C, D). TG/HDL-C ratio has been reported to be useful in predicting cardiovascular disease (19). It is surprising that 46.6% of patients with TG/ HDL-C ratio were above the average in excessive Aldo group, while only 26.5% in the normal group (Fig .1E). Our data also show that Aldo is not significantly correlative to LDL-C (Fig .1F). 

### HUVECs growth was synergistically inhibited with the combined treatment of Aldo and Ox-LDL

**Fig.2:**
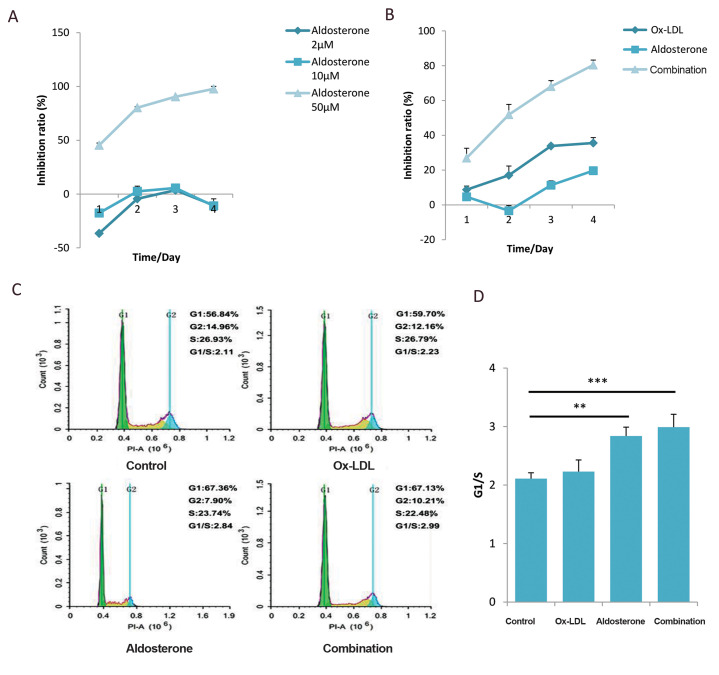
Aldo sterone and Ox-LDL block the cell cycle in the G1/S phase to inhibit proliferation of HUVECs. **A. **Cell growth curve of HUVECs under treatment of Aldo (2, 10 and 50 μM). **B. **Cell growth curve of HUVECs which were treated with 20 μM Aldo alone or combined with 120 μg/mL of Ox-LDL. At each time point, relative numbers of viable cells were detected using CCK-8 assay. **C. **Cell cycle was detected by flow cytometry after treatment with Aldo (20 μM), Ox-LDL (120 μg/mL) or Aldo plus Ox-LDL. **D.** G1/S phase ratio of HUVECs which were treated with different drugs. DMSO used as control. Data are presented as mean values ± standard errors from three experiments. **; P<0.005, ***; P<0.0005 compared with the control group, Ox-LDL; Oxidized low-density lipoprotein, HUVECs; Human umbilical vein endothelial cells, Aldo; Aldosterone, and DMSO; Dimethylsulfoxide.

Endothelial dysfunction plays a key role in AS (20). A common hallmark of these pathologic conditions is vascular dysfunction associated with endothelial cell growth inhibition, senescence, and death by apoptosis. HUVECs can be used as a cell model to understand further mechanisms involved in the endothelial dysfunction induced by the high level of Aldo and Ox-LDL. Our results showed that HUVECs growth can be inhibited by a high concentration of Aldo (Fig .2A). And cells growth was significantly inhibited even at a low concentration of Aldo when combined with the Ox-LDL together (Fig .2B). The cell cycle blockage may be the main reason for cell growth inhibition. Our results confirm that the cell cycle of HUVECs treated by Aldo and/or Ox-LDL can be blocked on the G1/S phase. The rate of G1/S increased significantly for the concomitant drugs group compared to the control group (Fig .2C, D). These data suggest that G1/S block is a major reason for inhibiting HUVECs growth, one kind of endothelial dysfunctions, which may be a mechanism for AS promoted by Aldo and Ox-LDL.

### Aldo accelerated senescence and promoted apoptosis in HUVECs induced by Ox-LDL

**Fig.3:**
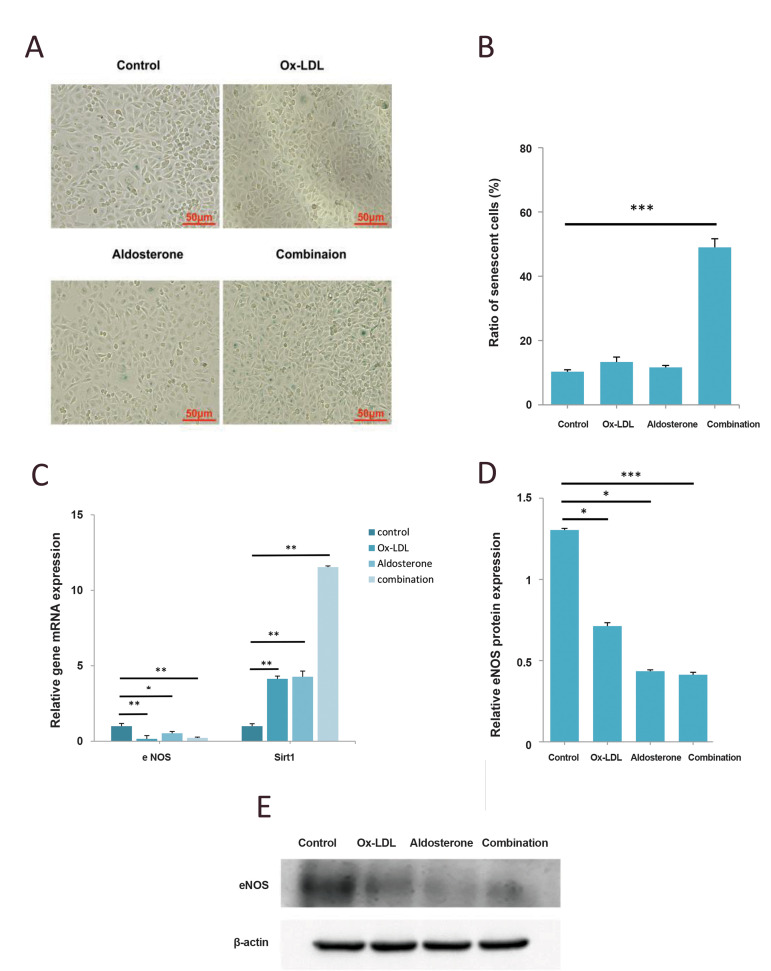
Aldo sterone accelerates senescence in HUVECs treated with Ox-LDL. HUVECs were treated with 20 μM Aldo alone or combined with 120 μg/mL of Ox-LDL for 48 hours. Cells were treated with the same amount of DMSO as control. **A.** Cell aging was detected by Senescence β-Galactosidase Staining Kit (scale bars: 50 μm).** B.** Ratio of SA-β galactosidase-positive HUVECs. **C-E. **Western blot and qPCR analysis of eNOS and Sirt1 in HUVECs. *; P<0.05, **; P<0.005, ***; P<0.0005, ****; P<0.0001 compared with the control group, HUVECs; Human umbilical vein endothelial cells, Ox-LDL; Oxidized low-density lipoprotein, DMSO; Dimethylsulfoxide, and qPCR; Quantitative polymerase chain reaction.

**Fig.4:**
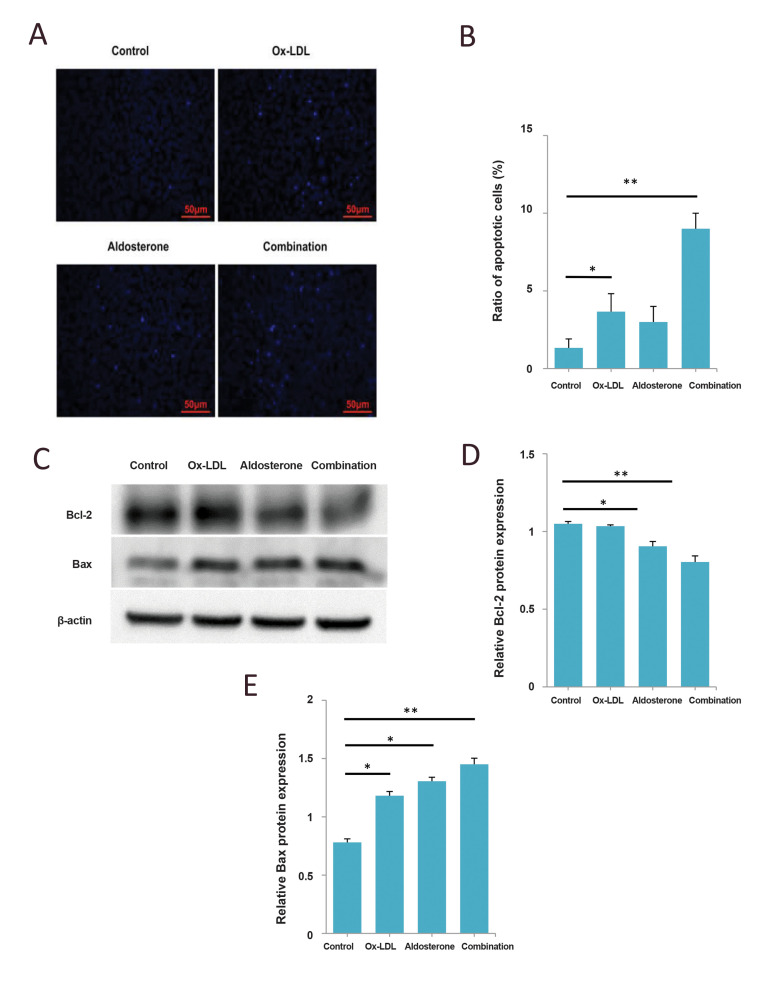
Aldo sterone promotes apoptosis in HUVECs induced by Ox-LDL. HUVECs were treated with 20 μM Aldo alone or combined with 120 μg/mL of OxLDL for 48 hours. Cells were treated with same amount of DMSO as control. **A.** The apoptosis of HUVECs was detected by Hoechst33342 staining (scale bars: 50 μm).** B.** Ratio of Hoechst33342 staining-positive HUVECs. **C-E.** Western blot analysis of Bcl-2 and Bax in HUVECs. β-actin is included as the loading control. Shown are mean values ± standard errors from three experiments. *; P<0.05, **; P<0.005 compared with the control group, HUVECs; Human umbilical vein endothelial cells, Ox-LDL; Oxidized low-density lipoprotein, and DMSO; Dimethylsulfoxide.

Cell aging analysis results show that the senescence of HUVECs has been accelerated by the combined treatment of Aldo and Ox-LDL, but almost was not affected by the single drug (Fig .3A, B). qPCR results showed that eNOS RNA levels dramatically decreased, and the Sirt1 mRNA levels significantly increased when HUVECs were treated with Aldo and Ox-LDL (Fig .3C). Western blot results confirmed that the pattern of the eNOS protein level is similar to the mRNA level (Fig .3D, E). Apoptosis assay results showed that the apoptosis rate of HUVECs significantly increased when combined treatment using both Aldo and Ox-LDL, compared with the control or single drug (Fig .4A, B). Western blot results suggested that the quantity of apoptosis-related critical protein Bcl2 decreased, and Bax increased in HUVECs treated with single/combined drugs (Fig .4C-E), which suggested that the apoptosis is mediated by Bcl-2 family proteins through the mitochondria-dependent pathway. These above key factors, including eNOS, Sirt, Bcl-2, and Bax, may be useful targets for the better prevention and therapy of AS.

**Fig.5:**
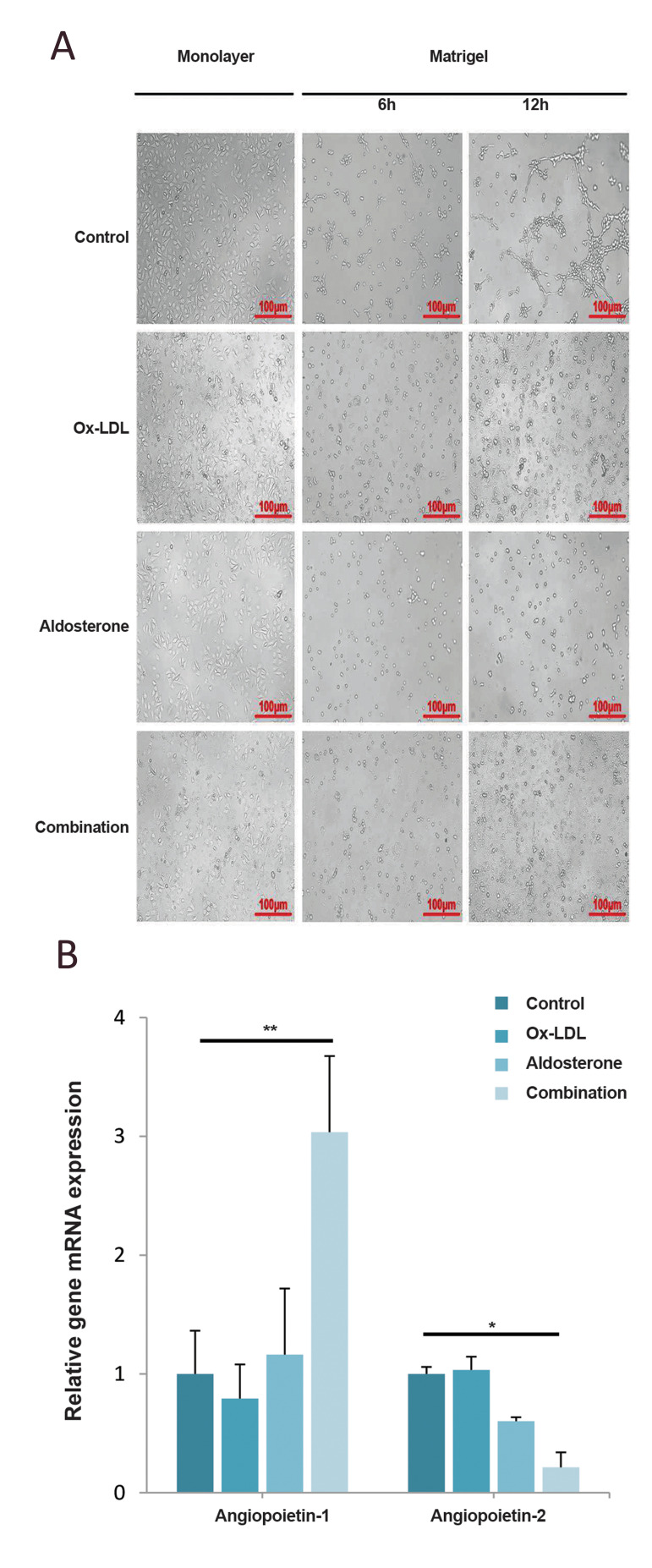
Aldo sterone and Ox-LDL inhibits tube formation* in vitro*. Cells were treated with Aldo (20 μM), Ox-LDL (120 μg/mL) or Aldo plus Ox-LDL for 48 hours, control was incubated with DMSO. **A. **Tube formation of HUVECs was examined by* in vitro* Matrigel tube formation assay (Scale bars: 100 μm). **B. **The relative expression of mRNA levels of Angiopoietin-1/2 in HUVECs was detected by qPCR. Shown are mean values ± standard errors from three experiments. *; P<0.05, **; P<0.005 compared with the control group, Ox-LDL; Oxidized lowdensity lipoprotein, DMSO; Dimethylsulfoxide, HUVECs; Human umbilical vein endothelial cells, h; hours, and qPCR; Quantitative polymerase chain reaction.

### Angiogenesis was inhibited in HUVECs treated with Aldo and/or Ox-LDL

Matrigel tube formation assay shows that angiogenesis of HUVECs was inhibited dramatically with the combined treatment of both Aldo and Ox-LDL (Fig .5A), which suggested that the ability to repair damaged cells attenuate. Both Angiopoietin1 and Angiopoietin2 are important in the process of Angiogenesis. qPCR results showed that Angiopoietin1 expression was promoted while Angiopoietin2 was inhibited in combined treatment (Fig .5B). The mechanism of angiogenesis regulated by Angiopoietin1/2 needs to be further clarified. 

## Discussion

Increasing evidence shows that Aldo plays crucial roles in the occurrence and progression of AS and promoting the formation of plaques (21). It is recognized that foam cell formation was a critical step, mainly according to the macrophages following exposure to Ox-LDL (22, 23). Patients’ statistical data analysis results showed that excessive Aldo is related directly with TG/HDL-C ratio, which is a main predictive factor for cardiovascular disease, and this elucidates that excessive Aldo level may cause AS by affecting both TG and HDL-C. It is useful to continue to collect the patient’s information.

An elevated level of plasma LDL-C is an important risk factor for AS. It is useful to decrease cardiovascular risk and prevent the progression of AS with controlling elevated LDL-C (24). Aldo has little effect on the LDL-C level in our patients. It is very interesting to investigate whether the progression of AS may be accelerated if Aldo affects the value of TG/HDL-C in the presence of a high level of LDL-C. There is a synergetic effect for the patient with excessive Aldo and Ox-LDL, which plays a central role in AS by acting on multiple cells, such as causing HUVECs in oxidative stress (25). Based on the HUVECs model, we found that cell growth and angiogenesis were synergistically inhibited. The rates of senescence and apoptosis were synergistically promoted when combined with both Aldo and Ox-LDL. Therefore, endothelial cells dysfunction, including growth, angiogenesis, senescence, and apoptosis, all of which were affected by Aldo in the presence of Ox-LDL. These findings provided more information for the clinical treatment of AS. 

Human vascular endothelial cell senescence and apoptosis are initiating factors in numerous cardiovascular diseases (26). Activation of the reninangiotensin-aldosterone (Aldo) system plays a critical role in endothelial dysfunction, vascular remodeling, and senescence (27). There are also many vital protein factors involved in the process of AS. eNOS/Sirt1 regulatory loops are the main factors in the ROS process, and Bcl2/Bax are the main factor involved in the cell apoptosis (28). Angiopoietin2 attenuates AS, and its up-regulation may have potential therapeutic value in patients with this disease (29). Our study showed that eNOS/Sirt1, the value of Bcl-2/Bax and Angiopoietin2 are significantly decreased with the concomitant treatment of Aldo and Ox-LDL. These factors play important roles in the process of endothelial cells dysfunction induced by Aldo and/or Ox-LDL. However, the mechanism of these key genes expression regulation by Aldo and Ox-LDL is still not clarified. 

In summary, HUVECs growth, angiogenesis, senescence, and apoptosis were significantly affected when treated with both Ox-LDL and Aldo. The further study of Aldo will provide a theoretical basis for the effective prevention and investigation of a new treatment of AS. High concentrations of Aldo and LDL-C may accelerate the process of the disease, especially for those patients with increased level of Aldo who must control their LDL-C. 
